# An overview of biomarkers in Alzheimer’s disease

**DOI:** 10.4103/0972-2327.74256

**Published:** 2010-12

**Authors:** Pandurang R Wattamwar, P. S. Mathuranath

**Affiliations:** Cognition & Behavioural Neurology Section, Department of Neurology, Sree Chitra Tirunal Institute for Medical Sciences & Technology (SCTIMST), Trivandrum, Kerala, India

**Keywords:** Alzheimer’s disease, biomarkers, biochemical, MRI volumetry, neuropsychology, positron emission tomography, tau

## Abstract

Alzheimer’s disease (AD) is the commonest progressive, dementing neurodegenerative disease in elderly, which affects innumerable people each year, and these numbers are likely to further increase as the population ages. In addition to the financial burden of AD on health care system, the disease has powerful emotional impact on caregivers and families of those afflicted. In this advancing era of AD research, with the availability of new treatment strategies having disease-modifying effects, there is growing need for the early diagnosis in AD, often hampered by paucity of biomarkers of AD. Various candidate biomarkers for AD have been developed that can detect patients with AD at an early stage. In the recent years, the search for an ideal biomarker has undergone a rapid evolution. Novel technologies in proteomics, genomics, and imaging techniques further expand the role of a biomarker not only in early diagnosis, but also in monitoring the response to various treatments. However, the availability of sensitive and specific biomarkers requires the method to be standardized so as to be able to compare the results across studies. Inspite of tremendous advances in this field the quest for an “ideal biomarker” still continues. In this review, we will discuss the various candidate markers in five spheres namely biochemical, neuroanatomical, metabolic, genetic and neuropsychological, and their current status and limitations in AD diagnosis.

## Introduction

Since the description of the “miliary bodies” and “dense bundles of fibrils” by Alois Alzheimer in 1907, in the brain of a woman with progressive dementing illness in her early 50s, amyloid plaques and neurofibrillary tangles (NFT) are now considered as pathological hallmarks of Alzheimer’s disease (AD).[1] It took nearly next 80 years to identify amyloid-β (Aβ) peptide, the main component of amyloid plaques,[2] and the amyloid precursor protein (APP), the source of Aβ.[3] Further research, particularly in the genetic domain, led to identification of APP and presenilin genes (*APP, PSEN1, and PSEN2*) and mutations in these genes as cause of rare forms of early-onset familial AD.[4,5] On other hand, ε4 allele of apolipoprotein E gene (APOE) has been recognized as a major risk factor for late-onset AD.[6] Later, insight into the molecular pathogenesis of AD came through many transgenic mouse models and tissue culture studies of AD which led to the proposition of the “amyloid cascade hypothesis”. According to this hypothesis, it has been proposed that accumulation of Aβ peptide is the upstream event in AD pathogenesis, in both early-onset and late-onset forms, leading to the formation of amyloid plaques, which trigger tau hyperphosphorylations and the formation of NFT, ultimately leading to synaptic dysfunction, neuronal loss, degeneration, and dementia.[7,8] However, there is growing evidence for the role of additional factors such as oxidative stress, neuroinflammation, and mitochondrial dysfunction in the pathogenesis of AD.[9]

This knowledge has rendered proposition of innovative treatment strategies, which are likely to have disease modifying potentials such as, active or passive Aβ immunotherapies, β- or γ-secretase inhibitors, and Aβ aggregation inhibitors, which are in various phases of clinical trial.[10] These disease-modifying agents will naturally be more effective when initiated in very early stage of AD (prodromal AD—minimal cognitive impairment [MCI] stage) or perhaps, if possible, even at asymptomatic stage (preclinical AD) of the disease, before formation and sufficient accumulation of amyloid plaques and NFT. Currently, clinical diagnosis of dementia is based on the criteria of the Diagnostic and Statistical Manual of Mental Disorders, fourth edition (DSM-IV-TR)[11] and of AD on the National Institute of Neurological Disorders and Stroke—Alzheimer’s Disease and Related Disorders Association (NINCDS–ADRDA)[12] working group criteria. As these diagnostic criteria are based on the appearance of clinical symptoms, the time when AD pathology has progressed sufficiently they fail to detect subjects at prodormal (MCI) or preclinical stage. Therefore, there is a growing need for the development of measures which can detect patients at an early stage. Various biomarkers in AD can at least partly serve this purpose. The biomarkers are the entities whose concentration, presence, and activity are objective evidence of a biological or pathogenic process. They can detect the patients with AD in their preclinical stage, monitor the disease progression, improve the understanding of various drug mechanisms targeting different pathogenic processes, and also detect treatment response more sensitively and objectively.

Hence, one of the most important goals of current research in AD is to develop and validate biomarkers which can detect at an early stage individuals who are likely to develop AD. Biomarkers in AD can be broadly classified in five spheres: biochemical, neuroanatomical, metabolic, genetic, and neuropsycological [Table 1]. In the present review, we will give an overview on the most extensively studied biomarkers for AD in these spheres, and their current status. In spite of advances in this field, the quest for an “ideal biomarker” [Table 2] still continues.[13]

**Table 1:** Various biomarkers in Alzheimer’s disease

Spheres	Biomarkers
Biochemical	CSF
	Blood-based
Neuroanatomical	CT scan
	MRI scan
Metabolic	PET scan
	SPECT scan
Genetic	
	APP
	PSEN1
	PSEN2
	APOE4
Neuropsychological	Episodic memory
	Other–attention, executive functioning, etc.

APP, Gene for amyloid precursor protein; APOE4, Apolipoprotein E4 allele; CSF, Cerebrospinal fluid; CT, Computed tomography; MRI, Magnetic resonance imaging; PET, Positron emission tomography; PSEN1 and PSEN 2, Preseniline gene 1 and 2; SPECT, Single photon emission computed tomography

**Table 2:** Criteria for an ideal biomarker[13]

Should detect a fundamental feature of the molecular pathogenesis or neuropathology of AD Should be validated in neuropathologically confirmed AD cases Should have a sensitivity >80% for detecting AD and a specificity >80% for differentiating AD from other dementias Should be able to detect AD in its early stages (i.e., during MCI) Be reliable, reproducible, noninvasive, simple to perform, inexpensive and, thus, adaptable in routine clinical practice

The main barriers for developing an ideal biomarker for AD is the lack of complete understanding of the underlying pathogenic process, unavailability of histopathological diagnosis during life, occurrence of large overlap with other types of dementia, especially dementia with Lewy bodies and vascular dementia and their influence on the different biomarkers, future progression of the disease and response to the available treatment.

## Biochemical Markers

In the last decades, various candidate biochemical markers in cerebrospinal fluid (CSF) as well as in peripheral blood have been evaluated through many cross-sectional and longitudinal studies and few in autopsy proven series of AD patients, for early diagnosis of AD.

## Cerebrospinal Fluid Biomarkers

Given the physiologic proximity of the CSF to the brain extracellular space, CSF still remains an attractive source of biomarkers for AD. For the same reason, it directly reflects the biochemical and molecular changes occurring within the interstitial environments of the brain parenchyma. Much of the initial attempts and research for a candidate biomarker in CSF were driven by “amyloid cascade theory” and hence were targeted towards the extracellular amyloid plaques and the intraneuronal NFT, the two pathological hallmarks of AD. However, with new insights into the role of various other pathogenic processes such as oxidative stress, mitochondrial dysfunction and so forth, more and more novel biomarkers are being evaluated.

CSF biomarkers for AD can be divided into “basic or nonspecific”, “core or specific”, and “novel biomarkers” [Table 3]. The nonspecific or basic biomarkers are useful to rule out important differential diagnoses. On the other hand, “core or specific biomarkers” reflect the central molecular pathogenesis of AD.

**Table 3:** Cerebrospinal fluid biomarkers in Alzheimer’s disease

Biomarkers	Pathogenic process	Changes seen in AD
Nonspecific or basic biomarkers		
CSF cell count	Inflammation	Normal
CSF:serum albumin ratio	BBB function	Normal
Intrathecal immunoglobulin synthesis	Inflammation	Normal
Specific or core biomarkers		
Total-tau	Neuronal injury	Marked increased (but not specific for AD)
Phosphorylated-tau	Neurofibrillary tangles	Marked increase (more specific for AD)
Aβ1-42	Amyloid plaque	Marked reduction
Novel biomarkers		
CSF BACE1	Amyloidogenesis	Increased
Truncated amyloid-β isoforms	Amyloidogenesis	Increased
APP isoforms	APP products	Increased
F2-isoprostane	Mitochondrial dysfunction	Increased
Biomarkers of synaptic degeneration	Synaptic dysfunction	Increased

### Basic or nonspecific cerebrospinal fluid biomarkers

Basic or nonspecific CSF biomarkers include cell counts, CSF:serum albumin ratio, and intrathecal immunoglobulin production and reflect the status of blood–brain barrier (BBB) and inflammatory processes in the brain. These biomarkers are neither deranged nor specific for underlying pathogenic process in AD, but serve the purpose of excluding various other AD mimickers including infections, inflammations, and vascular diseases.[14,15] For example, patients with vascular dementia usually have elevated albumin ratio, indicating impaired BBB function.[16]

### Core or specific cerebrospinal fluid biomarkers

The core or specific biomarkers reflect the underlying molecular pathology of AD especially, amyloid plaques, NFT, and axonal degeneration, and also are the most extensively studied biomarkers. Major component of extracellular amyloid plaque core is predominantly formed by 40- to 42-amino acid peptide (Aβ1-40 and Aβ1-42) derived by proteolytic cleavage of the larger transmembrane protein APP, expressed in both neural and nonneural tissues. A number of pathways for the processing of APP has been recognized.[17] Normally, in the nonamyloidogenic pathway α-secretase, by acting at the α-site, cleaves APP at N terminal, just above the surface of the cell membrane, producing soluble APP (sAPPα). This cleavage, being within the β-amyloid domain, precludes the formation of Aβ peptides. The C-terminal APP remnant is further digested by the transmembrane protein complex γ-secretase. On the other hand, in the amyloidogenic pathway, if APP is cleaved by β followed by γ-secretase at N- and C-terminals, respectively, it yields insoluble Aβ peptides.[18,19] The three most widely studied and established core CSF biomarkers include Aβ1-42, total tau (t-tau), phosphorylated tau (p-tau).

### Cerebrospinal fluid Aβ1-42

Many studies have shown that in patients with AD there is a reduction of Aβ1-42 by about 50% as compared with age-matched healthy nondemented controls with diagnostic sensitivity and specificity levels between 86 and 90%.[20,21] The correlation of low CSF Aβ1-42 levels and total amyloid load has also been shown in autopsy confirmed AD patients in different series.[22,23] It has been suggested that reduction in Aβ1-42 levels occurs secondary to aggregation and retention of Aβ peptides in the brain parenchyma as amyloid plaques. Positron emission tomography (PET) studies, utilizing novel Aβ ligands like Pittsburgh compound B (PIB), which enables direct visualization of the fibrillar Aβ load in the brain during life, have also shown that low CSF Aβ1-42 levels correlate inversely to the fibrillar amyloid load within brain parenchyma.[24–26] Therefore the available data further support that CSF Aβ1-42 levels reflect fibrillar Aβ1-42 levels and amyloid plaque load in the brain and with increasing amyloid plaque formation there is a progressive reduction in availability of Aβ to diffuse into the CSF.

### Cerebrospinal fluid total tau and phosphorylated tau

NFTs, the pathological hallmark of AD, consist of aggregated straight or paired helical filaments, twisted ribbons, or other conformations of aberrantly phosphorylated forms of the microtubule-associated protein (MAP) tau. The protein tau is an intracellular protein which maintains the stability of microtubules in neurons. In normal individuals, only low concentration of tau is present in CSF. Therefore, tau proteins may be considered promising candidate biomarkers for Alzheimer-type axonal degeneration and NFT formation.[27] Many studies have demonstrated an increase in the concentration of total tau (t-tau) in AD patients compared with nondemented elderly subjects, with the sensitivity and specificity levels between 80% and 90% respectively.[21,28] High CSF t-tau has also been associated with fast progression from MCI to AD as well as reflect the intensity of the disease.[29,30] However, the available data from various studies suggest that CSF t-tau levels just reflects the intensity of neuronal and axonal damage in the brain and is also elevated in patients with recent stroke or brain trauma as well as Creutzfeldt–Jakob disease (CJD).[31–33] Therefore t-tau alone may be not sensitive enough to differentiate patients with AD from other entities, especially vascular dementia and CJD. The sensitivity and specificity for diagnosis of AD improved when the combination of the two CSF markers (t-tau and Aβ1-42 levels) is used, however differentiating AD from other primary degenerative dementias was still unsatisfactory (sensitivity, 85%; specificity, 58%).[34]

It was later recognized that the NFT consists of hyperphosphorylated forms of the tau (p-tau) and concentration of p-tau protein in the CSF reflects the phosphorylation state of tau in the brain. Therefore, measuring p-tau levels in CSF may be more appropriate than t-tau alone. Approximately, 30 phosphorylation epitopes have been detected in AD. Most widely studied p-tau proteins are hyperphosphorylated at threonine 231 (p-tau_231P_) and at threonine 181 (p-tau_181P_). An increase in p-tau has consistently been found in the CSF of AD patients compared with controls with sensitivity and specificity levels of between 80% and 90%.[21] In addition, CSF levels of p-tau_231P_ have been found to correlate with not only NFT pathology in neocortex, but also the rate of hippocampal atrophy in the brain.[35,36] High CSF p-tau_181P_ has been associated with a fast progression from MCI to AD, and with rapid cognitive decline in AD.[35,36] As p-tau is not elevated in patients with acute stroke[31] or other neurodegenerative diseases such as CJD,[37] frontotemporal lobe dementia,[38] and normal pressure hydrocephalus,[39] in contrast to t-tau which just reflects intensity of axonal injury in these patients, the ratio between p-tau and t-tau is more helpful for identifying AD tau pathology and differentiating AD from these conditions.

Thus, studies have shown that core CSF biomarkers when used in combination are able to differentiate patients with AD from healthy elderly individuals, identify cases of prodromal AD in patients with MCI as well as can predict conversion to AD in the preclinical stage of the disease.

### Novel biomarkers

With the growing knowledge of underlying AD pathogenesis, biomarkers other than Aβ and tau are being described in various studies. These novel biomarkers are expected to further increase the sensitivity and specificity for the early diagnosis of patients with AD. We will discuss few of these novel biomarkers.

#### Cerebrospinal fluid BACE1

In the amyloidogenic pathway, APP is cleaved sequentially by β-secretase followed by γ-secretase, yielding insoluble Aβ peptides. The main enzyme responsible for β-secretase activity is β-site APP-cleaving enzyme 1 (BACE1). BACE 1 activity can be reliably detected within the brain and human CSF. An extracellular isoform of BACE1 found in CSF, is produced by membrane shedding. Studies have shown that BACE1 concentration and activity are increased in patients with AD, as well as in subjects with MCI (prodromal AD), as compared to controls.[40,41] In addition, the ApoE-ε4 genotype has been associated with increased BACE 1 activity in both AD and MCI subjects.[42]

#### Truncated amyloid-β isoforms

In addition to Aβ1-42, many shorter Aβ isoforms have been found in brain and in CSF of patients with AD, constituting a large family of peptides with variable lengths. These shorter peptides result from catalytic cleavage by BACE1 and γ-secretase at different positions in APP and/or involvement of other proteases. A few studies have shown that the measurement of Aβ42/Aβ40 or Aβ42/Aβ38 ratio might improve diagnostic accuracy in cases of AD than measuring Aβ1-42 alone.[43,44] However, further studies are needed to determine the clinical value of CSF measurements of detailed amyloid peptide patterns, as compared to CSF Aβ1-42 alone.

#### Amyloid precursor protein isoforms

Soluble N-terminal fragments of APP, the α-sAPP, and β-sAPP are the products of α-secretase or β-secretase induced cleavage, respectively. In sporadic AD and MCI, CSF levels of both α-sAPP and β-sAPP have been reported to remain unaltered.[45] However, these biomarkers might be valuable tools in clinical trials, especially for monitoring the drug effect.

#### F2-isoprostane

Recently, mitochondrial dysfunction has also been speculated as one of the pathogenic processes occurring in patients with AD. It is hypothesized that Aβ peptide after entering the mitochondria can induce generation of reactive oxygen species leading to mitochondrial dysfunction.[46] However, currently there is no CSF biomarker directly reflecting mitochondrial dysfunction, but the measurement of F2-isoprostane (F2-iP), a product of lipid peroxidation, which reflects oxidative stress in AD, has been shown to be elevated in patients with AD and MCI as compared to normal controls.[47,48] At present, as F2-iP measurements are not available widely, and it remains a scientific research tool.

#### Biomarkers of synaptic degeneration

In AD, synaptic dysfunction or loss occurs well before neurons die. Therefore, being an early and potentially reversible event in the AD pathogenesis, biomarkers for synaptic degeneration are valuable not only for early diagnosis, but also for monitoring disease progression and effects of novel drugs. Several candidate presynaptic and postsynaptic proteins which can be identified in CSF are being evaluated for this purpose. These include actin-associated protein arc, synaptotagmin, synapsin, synaptophysin, growth-associated protein (GAP-43), synaptosomal-associated protein 25, and neurogranin.[49,50]

#### Plasma biomarkers

Plasma-derived biomarkers would be the most ideal being least invasive and easy to obtain compared to CSF. The most extensively studied plasma biomarker is Aβ. The results of various cross-sectional studies are conflicting, few studies showing increased levels of Aβ1-42 or Aβ1-40 while others found no change, but some recent longitudinal studies have shown that low plasma Aβ1-42 or Aβ1-40 levels, or Aβ1-42/Aβ1-40 ratio may be markers of future cognitive decline.[51] These contradictory findings may be explained by the fact that major source of plasma Aβ are the peripheral tissues rather than brain. With the advent of proteomics, many candidate plasma biomarkers are being described in patients with AD as well as MCI including alpha-1-antitrypsin, complement factor H, alpha-2-macroglobulin, apolipoprotein J, and apolipoprotein A–I. However, the findings of these studies need further confirmation through longitudinal studies, nevertheless this is an ever advancing and promising research area.[52]

### Neuroanatomical markers

The major role of neuroimaging is not only for early diagnosis of AD, but also differentiating AD from the other forms of dementia In addition, it can be potentially used to predict the development of dementia in otherwise normally aging individuals as well as to monitor disease progression over time. Hence, it is considered as an important cognitive neuroscience research tool. Pathological changes leading to loss of synapses and neurons correlate with tissue atrophy, which can be detected by structural imaging. Structural imaging includes computer-assisted tomography (CT) which provides good spatial resolution and magnetic resonance imaging (MRI) which provides comparable spatial resolution with far better contrast resolution. However, in addition to better resolution, MRI has several advantages compared with CT, including optimal angulation of the imaging plane, no bone hardening artifacts in the temporal lobe region, excellent gray–white matter discrimination, and identification of additional vascular lesions, particularly small lacunes and white matter lesions. Here, we will discuss the role of MRI.

Atrophy of target structures can be estimated by various methods such as visual rating scales, hand-traced region of interest (ROI), semi-automated techniques such as voxel-based morphometry (VBM) or deformation-based morphometry and more recently fully automated techniques have been described which can measure regional or whole brain volume as well cortical thickness in various regions of interest. Each of these techniques have their own advantages and disadvantages.

Medial temporal lobe (MTL) structures being critical in memory, earliest AD-associated brain alterations occur in this brain region. Therefore, many volumetric studies measure specific MTL structures, most focusing on the hippocampus. Cross-sectional ROI studies have shown that the hippocampal and entorhinal volumes can reliably differentiate AD patients from normal elderly.[53] The absolute volumetric difference for the hippocampal volume between patients with amnestic MCI and normal elderly is approximately 7–11% and between mild-to-moderate AD and MCI is 19–39%. While the absolute volumetric difference for the entorhinal cortex between patients with amnestic MCI and normal elderly is 13–17% and between mild-to-moderate AD and MCI is 30–38%[54,55] Longitudinal ROI studies have shown that the annual atrophy of the hippocampus in patients with MCI and AD is much higher compared to that in healthy elderly subjects. Annual hippocampal atrophy rate for normal, MCI and AD is 1.6–1.7%, 2.8–3.7%, 3.5–4%, respectively, while that for the entorhinal cortex in AD is about 7%.[56]

VBM as compared to manual hand-traced ROI methods, permits faster and more reliable brain volume measurement and is being increasingly used not only for early diagnosis, but also to study AD progression. Recently, a characteristic pattern of regional brain atrophy has been reported during a period of 3 years prior to the diagnosis of AD, starting in MTL and spreading in posterior and anterior directions, in a temporospatial pattern similar to the spread of NFT.[57] These findings confirm that regional brain volume loss parallels the pathological processes in AD.

Using more automated MRI analysis that can assess volumes of different brain regions, it was shown that entorhinal cortex and supramarginal gyrus cortical thickness and hippocampal volumes could differentiate normal healthy subjects from MCI with 90%, and from AD with 100% specificity and sensitivity.[58] These results suggest that automated MRI measures can serve as noninvasive diagnostic markers for MCI and AD. Another study measuring cortical thickness with rapid automated method showed that it may help in early diagnosis of AD, up to 24 months before the clinical diagnostic criteria are met.[59]

### Metabolic markers

Molecular imaging by PET or single photon emission computed tomography (SPECT) with radiopharmaceutical agents, are routinely used as measures of metabolic activity in various parts of the brain. In addition, it allows us a quantitative evaluation of physiological functions and distribution of receptors with high sensitivity. 18F-fluorodeoxyglucose PET (FDG-PET) studies have been used to measure the cerebral glucose metabolism which indirectly indicates the level of synaptic activity. Thus, a decreased glucose uptake in the FDG-PET study is an indicator of impaired synaptic function. FDG-PET studies in patients with AD have shown specific topographic pattern of decreased metabolism in temporal-parietal, posterior cingulate, and precuneus distribution.[60,61] Greater decrease in the FDG uptake correlate with greater cognitive impairment along the continuum from normal to MCI to AD. A recent longitudinal FDG-PET study has shown that conversion of MCI to AD was associated with a faster decline of FDG uptake in two main areas, left anterior cingulate and subgenual region, thus emphasizing potential role of FDG-PET for monitoring early progression in AD.[62] In terms of diagnostic accuracy, PET studies have shown a high sensitivity (94%), but average specificity (73–78%) for the diagnosis of AD. When hippocampal hypometabolism in combination with decreased neocortical FDG uptake is considered, the specificity for the diagnosis of AD is further increased.[63] Similar specificity for diagnosis has been found with SPECT studying regional blood flow with Tc-hexamethylpropyleneamine oxime.[64]

With the advent of PIB a novel Aβ PET ligand, it is possible to directly visualize the fibrillar Aβ load in the brain during life. PIB PET studies, as discussed previously, show an inverse relation between *in vivo* amyloid imaging load and Aβ1-42 levels. In addition, a recent study has shown a strong relationship between PIB binding and the severity of memory impairment in patients with MCI, suggesting that individuals with increased cortical PIB binding are likely to progress to AD.[65] These findings not only imply the promising role of PIB-PET studies in detecting individuals at prodormal stage, but also suggest the potential for measuring the effects of an early intervention targeting amyloidogenesis. Many PIB-PET studies demonstrate a roughly twofold increase in tracer retention in patients with AD as compared to cognitively normal elderly individuals, while patients with amnestic MCI lie in an intermediate position.[66,67] Also, the topographical distribution of PIB retention matches that of regional fibrillar plaque distribution in these patients. However, more recent cross-sectional as well as longitudinal studies utilizing PIB-PET seem to suggest that the presence of brain amyloidosis alone is not sufficient to produce cognitive decline. Instead, the Aβ-induced neurodegeneration as noted by hippocampal atrophy on MRI is the direct substrate of cognitive impairment, implying a complimentary role for MRI and PIB imaging in AD.[66,67]

### Genetic markers

The genetics of AD are complex and not completely understood. In addition, heterogeneity of the disease is unambiguous because of the influence of mutations and polymorphisms in multiple genes together with nongenetic factors. Mutations causing early-onset familial AD (EOFAD) are transmitted in an autosomal-dominant fashion; these are less prevalent but are highly penetrant. On the other hand, increased risk for late-onset AD is associated with common polymorphisms which are highly prevalent, but have relatively low penetrance.

The first genetic defects leading to AD were identified in the APP gene (*APP*) causing early-onset familial AD. Since then, many different pathogenetic mutations have been identified in APP, all of which are missense mutations lying within or close to the domain encoding the Aβ peptide. However, when it was found that mutations in the APP gene are responsible for 5% or less of all early-onset familial AD, efforts were directed toward identifying other early-onset familial AD genes. Later presenilin 1 and 2 (*PSEN1; PSEN2*) were reported as novel early-onset familial AD genes on chromosomes 14 and 1, respectively.[5,6,68] PSEN1 encodes a highly conserved membrane protein that is required for γ-secretase activity. Mutations in this gene as well as those in APP and PSEN2 lead to an increase in Aβ42 levels, the primary component of amyloid plaques.

Late-onset AD is multifactorial and genetically more complex. Several genes and genetic polymorphisms have been suggested as AD susceptibility factors; however, the only well-verified susceptibility gene for AD is the APOE gene located on chromosome 19. The APOE gene encodes the apolipoprotein E protein, of which apolipoprotein E (APOE) ε4 allele is associated with increased risk of AD.[6] Several genome-wide linkage studies have nominated novel, potential susceptibility loci but results are inconsistent. A recent meta-analysis of linkage studies for AD found evidence for linkage on chromosomes 1, 7, and 8, suggesting that these loci may harbour susceptibility genes for late-onset AD.[69]

### Neuropsychological markers

Neuropsychological markers have been relatively fewer. Various studies have shown that older adults who later go on to develop AD perform more poorly across a broad range of neuropsychological measures when compared to older adults who remain asymptomatic.[70] These findings suggest that subtle impairment detected on neuropsychological evaluation may serve as a potential marker for early identification of individuals at risk of AD. Episodic memory loss is one of the earliest and most prominent features of preclinical AD, occurring several years before emergence of obvious cognitive and behavioral changes, which are typical of AD.[71,72] Therefore, episodic memory decline is considered as a strong predictor for future AD. This is supported by the fact that MTL and hippocampal formation, which are critical for episodic memory, are the early structures to be affected in patients with AD. Other domains which are most consistently associated with preclinical stage of AD are attention, executive functioning, processing speed, and language. Various studies have shown that either there is early impairment in these domains or significant differences are noticed between at-risk subjects and control subjects. The performance on the paired associate learning test, which is considered a sensitive marker of episodic memory, has been identified to be a good marker for early AD. On the other hand, global measures of cognition especially the mini-mental state examination (MMSE) are less consistently associated with preclinical AD.

### Combination biomarkers

It is obvious that no single biomarkers for AD satisfies all the criteria for an ideal biomarker and certain limitations are present in all. Given this limitation, the use of a combination of biomarkers may be needed to achieve accurate and reliable identification of preclinical AD, for example combination of CSF t-tau, p-tau and Aβ1-42, may be more reliable than a single marker. Alternatively, CSF biomarker may be combined with neuroanatomical, metabolic, neuropsychological, or genetic markers. Therefore, combined biochemical, imaging, cognitive and genetic assessments may be needed for this purpose.

## Role of Biomarkers in Clinical Trials

Making an accurate diagnosis of AD during the early stages of the disease is still a challenging task. Inclusion of a combination of biomarkers, like the presence of MTL atrophy on structural imaging, positive CSF biomarker, reduction in glucose metabolism in bilateral temporal parietal regions, an increase in binding of Aβ ligands, or the presence of genetic mutations causing familial AD in patients with MCI could be used as the inclusion criterion in clinical trials, and this combination may eventually increase the proportion of study subjects with underlying AD pathology, thus improving the likelihood of identifying more appropriately the beneficial drug effect. Many different treatment strategies are being evaluated in the clinical trials for AD. However, because of clinical, pathological, and genetic heterogeneity in AD, certain patients may be more responsive to certain treatment while others may less responsive. For example, patients with low CSF Aβ1-42 and high PIB retention might be more responsive to drugs which modify amyloidogenesis, while patients with elevated BACE1 levels to β-secretase inhibitors. Thus, the biomarkers can potentially be used not only to stratify the patients for different drug trials, but also for monitoring the drug effect more sensitively.

## Limitations of Biomarkers

In spite of major advances in the field of AD research especially for early detection of AD patients with a variety of modalities including biochemical, imaging, and genetic assessment one must understand the limitations of these markers. Most biomarkers are currently available as a research tool and not available yet in routine clinical practice. Furthermore, for biochemical marker study, guidelines for sample collection, storage, and transportation, all need to be evolved and followed meticulously since variation in them can affect the results. Different assay methodologies are available for biochemical markers, such as the enzyme-linked immunosorbent assay (ELISA) and the mulitparameter assays. The absolute values detected by these methods will also vary, and it is often difficult to compare the results obtained using different methodologies. Therefore, these assay method need to be standardized for comparability or correction factor needs to be introduced to compare the results of different studies. There is, thus, still a lack of efficacious, less invasive and potentially less costly peripheral blood-based markers which can detect patients with AD with high sensitivity and specificity. Future studies should be directed to addressing this area. Many novel candidate biochemical biomarkers have been proposed for early diagnosis of AD, unfortunately, to date the validity of these markers is still suboptimal.

Neuroanatomical markers utilizing structural imaging, especially the manual methods for measuring regional brain volumes, have high accuracy, but are operator-dependent requiring proficient and knowledgeable operators and are also time consuming and painstaking. On the other hand, more automated MRI methodologies are highly sophisticated but require access to special software and sophisticated manual preprocessing prior to automated analysis. Future studies should be directed at validating simpler, more efficient methods for clinical use. PET imaging is still not widely available, especially in developing countries and does not measure a specific disease mechanism or treatment target. Its utility is further hampered by high cost. The optimal method for quantifying brain amyloid load is still not clear, the PIB-PET being most widely studied. However, a significant overlap is seen in PIB retention pattern among cognitively normal elderly and those with MCI or AD. In addition, the short half-life of 11C limits its use to major research centers, signifying the need for the development of more stable tracers.

Other than three disease-causing genes (*APP, PSEN1*, and *PSEN2*) and a single susceptibility gene (*APOE4*) which are firmly established genes related to AD, till date, the role of other putative genetic loci in the AD risk remains to be firmly established. However, some of these loci exhibit genetic linkage and/or association with AD across independent datasets and therefore are worthy of further investigation.

## Concluding Remarks

Significant advances have been made towards identifying patients with AD at an early stage or even at preclinical stage, which is the emergent need at this phase of AD research, where increasingly novel treatment strategies are being evaluated through clinical trials. Various biomarkers for AD are valuable tools in this regard, not only for identifying cognitively healthy individuals, who might develop AD or diagnosing AD at MCI stage (prodormal stage), but also for monitoring the progression in patients with AD. Ultimately, a combination of various biomarker rather than a single one may serve the purpose more advantageously. However future studies using postmortem pathological confirmation are needed to validate the novel markers or a combination thereof that will best predict, who will develop AD. Once this is achieved, it may become possible to move on to the next phase of developing truly preventive strategies.
